# High BMI and Insulin Resistance Are Risk Factors for Spontaneous Abortion in Patients With Polycystic Ovary Syndrome Undergoing Assisted Reproductive Treatment: A Systematic Review and Meta-Analysis

**DOI:** 10.3389/fendo.2020.592495

**Published:** 2020-12-03

**Authors:** Yi-Fei Sun, Jie Zhang, Yue-Ming Xu, Zi-Yu Cao, Yi-Zhuo Wang, Gui-Min Hao, Bu-Lang Gao

**Affiliations:** Department of Reproductive Medicine, The Second Hospital of Hebei Medical University, Shijiazhuang, China

**Keywords:** polycystic ovary syndrome, spontaneous abortion, body mass index, insulin resistance, assisted reproductive treatment

## Abstract

**Background:**

The risk of spontaneous abortion in patients with polycystic ovary syndrome (PCOS) undergoing assisted reproductive treatment (ART) is higher than that in patients without PCOS, however, no definitive risk factors have been confirmed to associate with the high spontaneous abortion rate in PCOS patients undergoing ART. This study was performed to assess the impact of relevant risk factors on spontaneous abortion in patients with PCOS. Clinical questions were formulated and organized according to the PICOS principle.

**Methods:**

A systematic review and meta-analysis were conducted on all published studies on PCOS and spontaneous abortion in Embase, PubMed, Web of Science and Cochrane Library. Related risk factors included body mass index (BMI), age, insulin resistance (IR), hyperandrogenism, and chromosome aberrations. All patients were diagnosed as PCOS using the Rotterdam criteria. The primary endpoint was miscarriage and live birth rate. Fixed-effect models were used to analyze homogeneous data, and subgroup and sensitivity analyses were performed on heterogeneous data. The source of heterogeneity was evaluated, and the random effect model was used to summarize the heterogeneity.

**Results:**

Among 1836 retrieved articles, 22 were eligible and included in the analysis with 11182 patients. High BMI (OR = 1.48, 95% CI [1.32, 1.67], MD = 1.35, 95% CI [0.58,2.12]) and insulin resistance (MD = 0.32, 95% CI [0.15, 0.49]) were associated with an increased risk of spontaneous abortion in PCOS patients undergoing ART. Older age (OR = 0.29, 95% CI [0.29, 0.44], MD = 2.01, 95% CI [0.04, 4.18]), embryonic chromosomal aberrations (OR = 0.75, 95%CI [0.31,1.77]), and hyperandrogenism (MD = 0.10, 95% CI [- 0.02, 0.22]) were not associated with the high spontaneous abortion rate in patients with PCOS. A subgroup analysis of BMI showed that there was no statistically significant difference in the effect between overweight and obesity on spontaneous abortion in PCOS patients undergoing ART (OR = 1.34, 95% [0.97, 1.85]).

**Conclusion:**

High BMI and insulin resistance are two risk factors for an increased risk of spontaneous abortion in PCOS patients undergoing ART, and losing weight and mitigating insulin resistance may decrease the spontaneous abortion rate in these patients undergoing ART.

## Introduction

Polycystic ovary syndrome (PCOS) is a complex endocrinopathy. According to the Rotterdam criteria, two of the three criteria had to be met to fit the definition of PCOS: chronic anovulation, clinical and/or biochemical evidence of hyperandrogenism, and polycystic ovaries (1). After the Endocrine Society Clinical Practice Guideline has suggested use of the Rotterdam criteria for diagnosing PCOS (2), the Rotterdam criteria has become the most widely used PCOS diagnostic standard in the world.

The characteristics of PCOS are follicular dysplasia, insulin resistance, and hyperandrogenism, affecting 5–10% women in childbearing age (3, 4). Due to abnormal endocrine changes, it is often associated with obesity, amenorrhea, hairiness, infertility, and most importantly, miscarriage. Previous meta-analyses (5, 6) have reported that women with PCOS have an increased risk of miscarriage compared to those without PCOS. In addition, patients with PCOS usually have a high abortion rate of 30–50% in the first 3 months of pregnancy, a high incidence of recurrent early abortion of 36–82%, and a high incidence of habitual abortion of 58% (7). Recurrent miscarriage was defined as two or more consecutive abortions with the same sexual partner (8). At this time, the patient has suffered at least 2 pregnancy losses. The related risk factors of recurrent abortion include chromosomal abnormalities, uterine abnormalities, antiphospholipid syndrome, obesity (9), and high risk of thrombosis (10). Among them, the recurrent abortion caused by abnormal chromosome can reach 60.6% (9). Spontaneous abortion is defined as pregnancy loss before 20 weeks of pregnancy (11). It has been found that the frequency and distribution of chromosomal abnormalities in the spontaneous abortion group are different from those in the recurrent abortion group (12). Moreover, abnormalities in the endocrinology, immunology and anatomy also play different roles in both groups (13). The pregnancy loss rate in natural conception was reported to be 10–16% (14, 15). High rates of early pregnancy loss, ranging from 12 to 48%, have been reported in assisted reproductive treatment (ART) (16, 17). Maternal age, controlled ovarian hyperstimulation protocol, cycle type, and PCOS status (18) may have an impact on the miscarriage rate (19). However, ART is a choice that some PCOS patients have to face in order to get pregnant. If the systematic risk factors can be found for spontaneous abortion in patients with PCOS undergoing ART, the pregnancy conditions can be improved before the first pregnancy so as to prevent pregnancy loss and economic loss in PCOS patients.

However, no specific meta-analyses have been conducted to analyze the risk factors for an increased risk of spontaneous abortion in patients with PCOS. These risk factors may include body mass index (BMI), age, hyperandrogenism, insulin resistance, and chromosome aberrations, which can be detected by observational studies on patients with or without these factors (20–42). Nevertheless, it is still unclear whether the above mentioned risk factors were comprehensive and whether they could cause an increase in the spontaneous abortion rate in PCOS patients undergoing ART. For instance, controversies exist regarding the role of obesity or overweight in adverse pregnancy outcomes in PCOS patients, with some authors (20, 36) considering obesity as a risk factor for adverse pregnancy outcomes while others (35) finding that BMI had no adverse effects on the pregnancy outcome. In view of these controversies, we believed that a meta-analysis was necessary to evaluate the risk factors of spontaneous abortion in PCOS patients undergoing ART in order to provide recommendations for clinical treatment of PCOS. In order to display all the viewpoints in a comprehensive and balanced manner, we decided to objectively search all documents related to PCOS and abortion. We selected the literature that reported spontaneous abortion after ART and studied possible risk factors for analysis.

## Methods

This study was performed in accordance with the preferred reporting items for systematic reviews and meta-analyses (PRISMA) statement (43). The PRISMA Checklist is shown in **Supplementary Table 1**. The protocol had been submitted to the International System Evaluation Expected Register (PROSPERO) with the registration number of CRD42020171499. The clinical reasoning was broken down and organized according to the PICOS principle.

### Search Strategy and Selection Criteria

We selected the search keywords PCOS and abortion, covering all the subject terms and free words under the classification of PCOS and abortion. The search database included Embase, PubMed, Web of Science and Cochrane Library (including Cochrane Database of Systematic Reviews, Database of Abstracts of reviews of effects, Cochrane Central Register of Controlled Trials, Cochrane Methodology Register, Health Technology Assessments database, NHS Economic Evaluation Database and About the Cochrane collaboration). Citation retrieval and manual retrieval were also performed to ensure that the largest number of relevant documents could be retrieved. Unpublished articles such as conference proceedings were also included. Relevant observational studies published from January, 1970 to March, 2020 were searched with no language restrictions. The search strategy is shown in **Supplementary Table 2**. The electronic search and the eligibility of the studies were independently assessed by two of the authors (Y-FS and Z-YC),

### Study Selection and Data Extraction

Observational studies with either a cohort study or case-control design in women with PCOS were selected. The inclusion criteria were articles evaluating spontaneous abortion in women with PCOS diagnosed according to the Rotterdam criteria (Revised 2003 criteria1: chronic anovulation, clinical and/or biochemical signs of hyperandrogenism, polycystic ovaries and exclusion of other etiologies (congenital adrenal hyperplasia, androgen-secreting tumors, and Cushing’s syndrome), and parameters which were associated with abortion in PCOS patients including age, BMI, and hyperandrogenism (1). The exclusion criteria were articles that did not use the Rotterdam criteria for diagnosis of PCOS, that included research subjects who had a history of recurrent abortion, that involved drug administration (including metformin) or intervention for purposes other than assisted reproductive technology (ART), and that were case reports, case series, or reviews (**Supplementary Figure 1**). Two authors independently studied the titles, abstracts, and full text for inclusion. In disagreement, a third physician was involved to reach an agreement. The following data were extracted from each selected study: author name, year of publication, study design, study location, participants’ characteristics (such as race, age, and BMI), and number of spontaneous abortion or ongoing pregnancy in patients with PCOS. All information was entered into a researcher‐developed data extraction form.

All included studies were assessed for risk of bias using the Newcastle‐Ottawa Scale (NOS) for non‐randomized studies (**Supplementary Table 3–4**). The NOS checklist contains three parameters of quality: (i) selected population, (ii) comparability of groups, and (iii) assessment of either the exposure or outcome of interest for case-control or cohort studies (44). Individual items assessed according to the NOS included representativeness of miscarriage and ongoing pregnancy groups in PCOS patients undergoing ART, ascertainment of diagnostic criteria for PCOS, pregnancy and delivery outcomes, cohort comparability on the basis of design or analysis, reliability of the results obtained, and adequate follow-up time. The quality of studies was assessed with the maximal stars of nine, and the studies were ranked as poor if there were less than five stars and good if there were five or more than five stars (45).

### Statistical Analysis

The Review Manger (version 5.3, The Cochrane Collaboration, Copenhagen, Denmark) was used for all statistical analyses. According to the results of literature search, the associations of the spontaneous abortion rate in PCOS patients undergoing ART were evaluated with the risk factors of BMI, age, hyperandrogenism, insulin resistance, and chromosome aberrations (6). Hyperandrogenism, BMI, age, and insulin resistance were treated as continuous variables and presented as mean difference (MD) and 95% confidence interval (CI) between exposed and control groups. If the 95% CI does not include 0, the study is statistically significant, indicating that this indicator is an influencing factor affecting the spontaneous abortion rate in patients with PCOS. If the MD is >0, this factor is a risk factor; otherwise the factor is a protective factor. The age, BMI, and chromosome aberrations were also analyzed as dichotomous variables and presented with odds ratio (OR) and 95% CI between exposed and control groups. If the 95% CI does not include 1, the study is statistically significant, indicating that this indicator is an influencing factor affecting the spontaneous abortion rate in patients with PCOS. If the OR is >1, this factor is a risk factor; otherwise the factor is a protective factor. The homogeneity of effect size across studies was tested by Q statistics, and the I^2^ statistics was used to measure the inconsistency of risk factors’ effects across studies, with I^2^ of 0–24% indicating slight heterogeneity, 25-49% moderate heterogeneity, 50–75% substantial heterogeneity, and over 75% considerable heterogeneity (46). If the article was homogeneous (P<0.1 or I^2^>25%), the fixed effect model (Mantel-Haenszel method) was chosen to test the additional uncertainty associated with the risk factors of different abortion rates in patients with and without PCOS. Otherwise, the random-effect model (DerSimonian–Laird method) was preferred. The possibility of publication bias was assessed by constructing a funnel plot.

## Results

### Search Results

A total of 1,843 studies were retrieved according to the search strategy. A total of 54 articles were excluded at the full text stage, including 8 articles which did not use the Rotterdam criteria for the diagnosis of PCOS, 13 reviews, 11 non-clinical trials, 6 about recurrent spontaneous abortion, 12 which were not about miscarriage related risk factors, and 4 which did not have complete data. Finally, 22 studies were chosen for the analysis (**Supplementary Figure 1**). These 22 studies were published between 2006 and 2019 (14 of which were published in the last three years) (**Supplementary Table 5**).

### Characteristics of Included Studies

Outcomes of interest were reported in 11182 patients in 17 retrospective cohort studies (20–26, 28, 30–36, 40, 41), three prospective cohort studies (38, 39, 42) and two case-control studies (27, 37). Fourteen studies were conducted in China (22, 23, 25, 28, 30–33, 35, 36, 38, 39, 41, 42), five in the USA (20, 21, 24, 26, 27), and the remaining three in Japan (37), Turkey (34), and Australia (40). Seventeen studies measured BMI (20–26, 30–32, 34–38, 41, 42), four assessed age (28, 30, 37, 40), three evaluated insulin resistance (30, 37, 42), five investigated chromosome aberrations (26, 30, 31, 33, 39), and the other four assessed hyperandrogenism (27, 30, 37, 42).

### BMI

**Figure 1:**
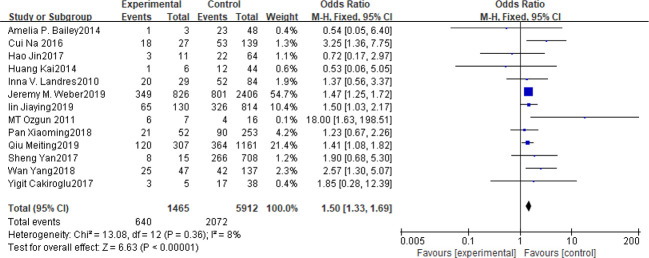
Meta‐analyses for BMI in categorical data.

**Figure 2:**
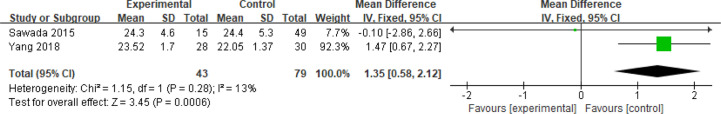
Meta‐analysis for BMI in continuous data.

**Figure 3:**
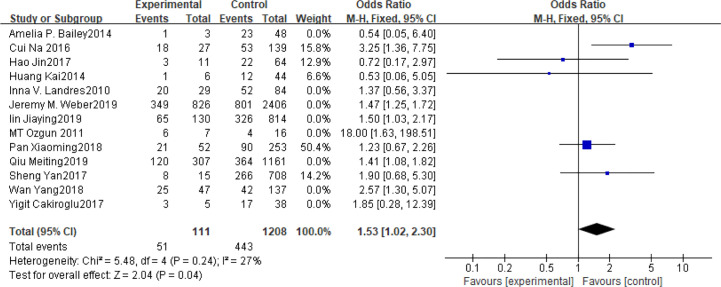
Meta‐analyses for BMI with Chinese BMI standard in categorical data.

**Figure 4:**
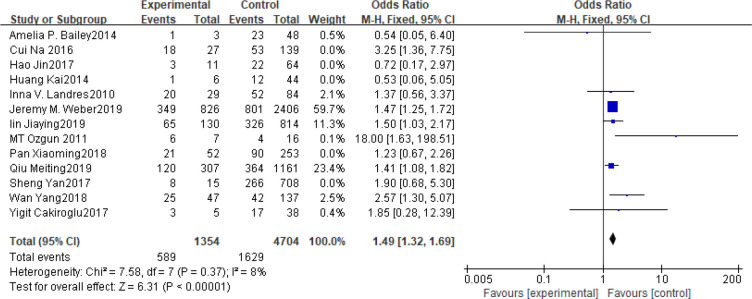
Meta‐analyses for BMI with WHO BMI standard in categorical data.

**Figure 5:**
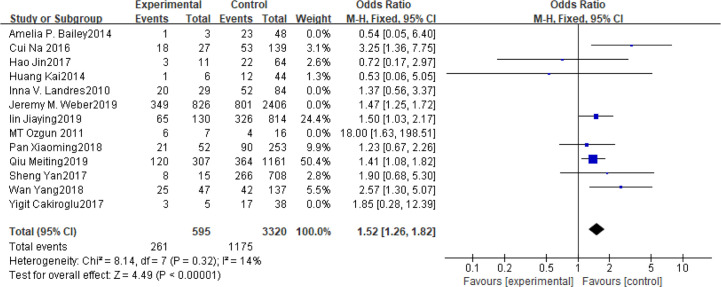
Meta‐analyses for BMI in Chinese in categorical data.

**Figure 6:**
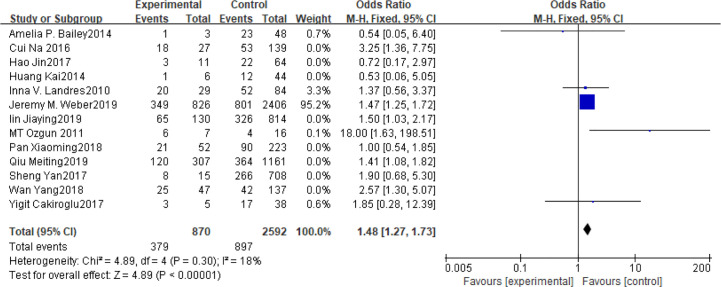
Meta‐analyses for BMI in non-Chinese in categorical data.

Fifteen articles with a total of 7499 patients evaluated the relationship between spontaneous abortion and BMI in PCOS patients undergoing ART (20–26, 32, 34–38, 41, 42), including 13 articles with categorical variables (20–26, 32, 34–36, 38, 41) and two with continuous variables (37, 42). Assessment of the risk of bias demonstrated a symmetrical funnel chart, indicating no publication bias (**Supplementary Figure 2**). The BMI was defined as weight in kilograms divided by the square of height in meters. In order to better show the homogeneity in heterogeneity analysis, patients were divided into overweight and obesity based on the BMI of different races. Among 13 articles with two categorical variables, eight were from China (22, 23, 25, 32, 35, 36, 38, 41), five of which used the Chinese BMI standard (47) of BMI ≥ 24 kg/m^2^ as overweight (22, 23, 25, 35, 38), while the remaining three used the WHO BMI standard (48) of BMI ≥ 25 kg/m^2^ as overweight (32, 36, 41). All the others studies applied the standard of BMI ≥ 25 kg/m^2^ as overweight (20, 21, 24, 26, 34). All these included articles showed a good homogeneity (I^2^ = 8% for categorical variables and I^2^ = 13% for continuous variables). The fixed effect model analysis showed that PCOS patients who had a high BMI were associated with a high rate of spontaneous abortion (OR = 1.48, 95% CI [1.32, 1.67], MD = 1.35, 95% CI [0.58, 2.12]) (**Figures 1** and **2**), However, different ethnic BMI standards may lead to selection bias and inaccurate results. In order to solve the problems of possible heterogeneity and selection bias, we conducted a subgroup analysis of the included literature: first, subgroup analysis was further performed according to the BMI standard (OR = 1.53, 95% CI [1.02, 2.30] for Chinese BMI standard, and OR = 1.49, 95% CI [1.32, 1.69] for WHO BMI standard) (**Figures 3** and **4**). The results of the two-subgroup analysis were consistent with the overall results, that is, high BMI was a risk factor for spontaneous abortion in patients with PCOS, indicating that different BMI standards did not affect the final results. Secondly, subgroup analysis was further performed according to the race (OR = 1.52, 95% CI [1.26, 1.82] for Chinese, and OR = 1.48, 95% CI [1.27, 1.73] for non-Chinese) (**Figures 5** and **6**). The subgroup analysis results of different races were consistent with the overall results, indicating that the BMI standard was applicable to the selected races and did not affect the final results.

**Figure 7:**
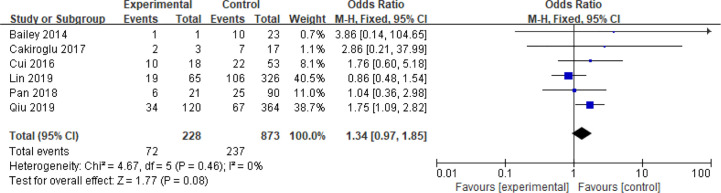
Meta‐analysis for overweight and obesity in categorical data.

In addition, six articles further divided PCOS patients with high BMI into overweight and obese subgroups (20–22, 32, 35, 36), two of which used the Chinese standard (47) of BMI ≥ 28 kg/m^2^ as obese and BMI 24–28 kg/m^2^ as overweight (22, 35), while the remaining four used the WHO standard (48) of BMI ≥ 30 kg/m^2^ as obese and BMI 25–30 kg/m^2^ as overweight (20, 21, 32, 36). Analysis of these two subgroups did not show any significant differences in the spontaneous abortion rate caused by obesity compared with overweight (OR = 1.34 95% [0.97, 1.85]) even though these articles were homogeneous (I^2^ = 0%) (**Figure 7**).

### Age

**Figure 8:**
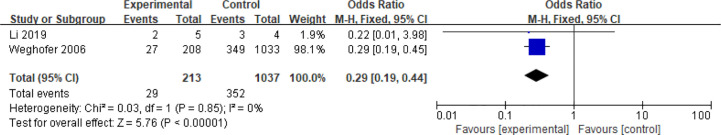
Meta‐analysis for age in categorical data.

**Figure 9:**
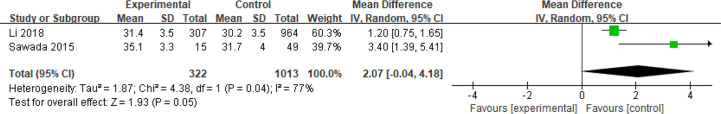
Meta‐analysis for age for continuous data.

Four articles with a total of 2585 patients focused on the relationship between age and spontaneous abortions in PCOS patients undergoing ART (28, 30, 37, 40). Among them, two articles reported dichotomous data (28, 40), while the other two reported continuous variables (30, 37). People over 35 years old were considered senior in one study (28), and 38 years of age was the line for senior in another (40). Age was demonstrated to be a protective factor for spontaneous abortions in patients with PCOS (OR = 0.29, 95% CI [0.29, 0.44]) in the two articles with dichotomous variables which had good homogeneity (I^2^ = 0%) (**Figure 8**). However, age was not related to the spontaneous abortions rate in PCOS patients undergoing ART (MD = 2.01, 95% CI [0.04, 4.18]) in the articles with continuous variables (**Figure 9**).

### Insulin Resistance

**Figure 10:**
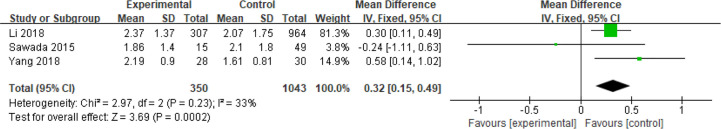
Meta‐analysis for insulin resistance.

The relationship between insulin resistance and spontaneous abortion in PCOS patients undergoing ART were examined in a total of 1393 patients in three reports (30, 37, 42). The homeostasis model assessment‐insulin resistance (HOMA ‐ IR) was obtained from the following equation: HOMA ‐ IR = fasting plasma insulin [mIU/L] × fasting plasma glucose [mmol/L]/22.5 (49) or HOMA - IR = fasting glucose level (mg/dL) ×fasting insulin level (µU/mL)/405 (37). The effects of HOMA‐IR on parameters and outcomes were analyzed, and it was shown that the IR increased the risk of spontaneous abortions in patients with PCOS (MD = 0.32, 95% CI [0.15, 0.49]) (**Figure 10**).

### Chromosome Aberrations

**Figure 11:**
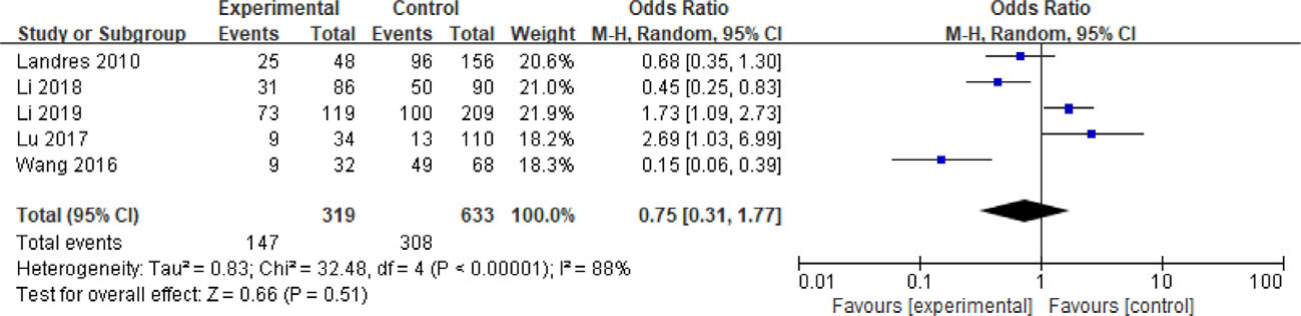
Meta‐analysis for Chromosome Aberrations.

**Figure 12:**
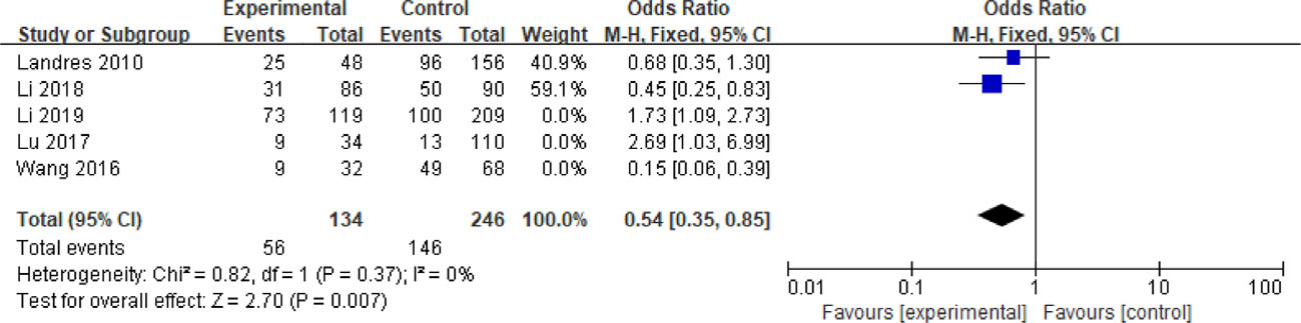
Meta‐analysis for G-banded chromosome karyotype analysis.

**Figure 13:**
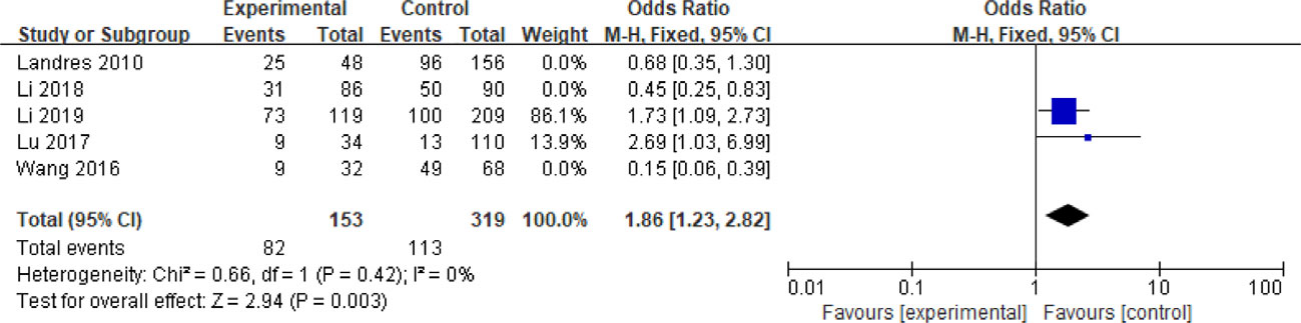
Meta‐analysis for SNP-array analysis.

Five articles with a total of 952 patients with dichotomous variables reported the relationship between embryo chromosomal abnormalities and spontaneous abortion in patients with PCOS undergoing ART (26, 30, 31, 33, 39). The probability of embryonic chromosome aberrations in abortion was not significantly increased in these PCOS patients (OR = 0.75, 95% CI [0.31, 1.77]) (**Figure 11**), indicating that the embryo chromosomal abnormalities was not a risk factor for spontaneous abortion in patients with PCOS. Further analysis of these articles revealed that the two studies by Landres et al. (26) and Li et al. (30) used G-banded chromosome karyotype analysis to detect chromosomal abnormalities, whereas the two studies by Li et al. (31) and Lu et al. (33) used SNP-array analysis. The remaining one article applied the traditional karyotyping combined with MLPA subtelomere assay, FISH analysis or ArrayCGH analysis to diagnose chromosome aneuploidy (39). Analysis of the above two pairs of studies found that these articles were homogeneous (both I^2^ = 0%) (**Figures 12** and **13**). The G-banded chromosome karyotype analysis did not show a significant increase in the probability of chromosome aberrations in embryos of patients with PCOS (OR = 0.54, 95% CI [0.35, 0.85]) (26, 30), whereas the SNP array analysis demonstrated the embryo chromosomal abnormality to be a risk factor for increased abortions in patients with PCOS (OR = 1.86, 95% CI [1.23, 2.82]) (31, 33).

### Hyperandrogenism

**Figure 14:**
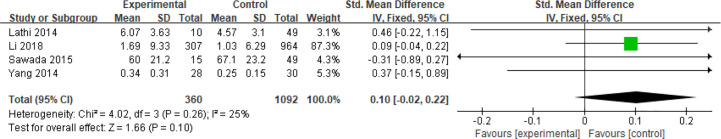
Meta‐analysis for hyperandrogenism.

Four articles including a total of 1,452 patients with continuous data assessed the effect of hyperandrogenism on spontaneous abortions in PCOS patients undergoing ART (27, 30, 37, 42). The 2003 Rotterdam criteria did not clearly specify the standard range of androgen values in hypersexual hormones. A study according to the Rotterdam criteria defined hyperandrogenemia as the finding of elevated androgens, and the specific cutoff values for testosterone, dehydroepiandrosterone sulfate (DHEAS) and Δ4 were 65 ng/dL, 2,800 μg/L and 2.5 ng/mL, respectively (50). Due to the differences in measurement methods in the included articles, the Standard Mean Difference (SMD) was chosen as the effect size indicator for analysis. The SMD could eliminate not only the influence of the absolute value but also the influence of the unit on the result. Analysis of these articles which had moderate heterogeneity (I^2^ = 25%) revealed that hyperandrogenism did not increase the risk of miscarriage in patients with PCOS (MD = 0.10, 95% CI [- 0.02, 0.22]) (**Figure 14**).

### Basal Luteinizing Hormone

**Figure 15:**
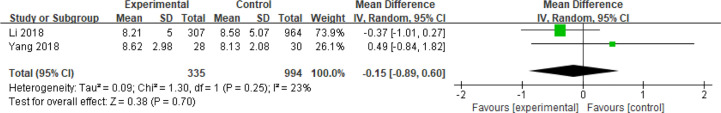
Meta‐analysis for basal luteinizing hormone.

Two articles enrolling a total of 1329 patients with continuous data assessed the effect of basal luteinizing hormone (bLH) on spontaneous abortions in patients with PCOS (30, 42). Analysis of these articles which had low heterogeneity (I^2^ = 23%) revealed that bLH did not increase the risk of miscarriage in patients with PCOS (MD = -0.15, 95% CI [- 0.89, 0.60]) (**Figure 15**).

### Adjustment of Confounding Factors

The above data were from the original data of the included literature, and the risk factors had not been adjusted. However, there were mutual influences between risk factors. In order to eliminate the influence of possible confounding factors, we analyzed the extracted adjusted OR value. Unfortunately, only two articles (30, 42) had adjusted the confounding effects of BMI, age, bLH, basal testosterone (bT) levels, and number of oocytes retrieved on spontaneous abortion in PCOS patients. The results showed that BMI (OR = 1.27, 95% CI [1.17,1.38]) was a risk factor for spontaneous abortion in PCOS patients undergoing ART, whereas age (OR = 1.06, 95% CI [0.95, 1.19]), bLH (OR = 1.01, 95% CI [0.95, 1.08]), and bT (OR = 1.10, 95% CI [0.70, 1.73]) had no effect on spontaneous abortion in PCOS patients. This was consistent with the conclusion obtained with the original data (**Supplementary Figure 3**).

## Discussion

In this systematic review and meta-analysis, it was found that high BMI (OR = 1.48, 95% CI [1.32, 1.67], MD = 1.35, 95% CI [0.58, 2.12]) and insulin resistance (MD = 0.32, 95% CI [0.15, 0.49]) were associated with an increased risk of spontaneous abortion in patients with PCOS undergoing ART. Older age (OR = 0.29,95% CI [0.29, 0.44], MD = 2.01, 95% CI [0.04, 4.18]), embryonic chromosomal aberrations (OR = 0.75,95%CI [0.31,1.77]) and hyperandrogenism (MD = 0.10, 95% CI [- 0.02, 0.22]) were not associated with higher spontaneous abortion rates in PCOS patients with undergoing ART.

This meta-analysis found that PCOS patients with spontaneous abortion history showed higher BMI than those with ongoing pregnancy (OR: 1.71, 95% CI [1.34, 2.19]). The results of subgroup analysis of overweight and obesity under different standards (i.e., under the Chinese BMI standard with the BMI ≥ 28 kg/m2 as obese and BMI 24–28 kg/m2 as overweight; under the WHO standard with the BMI ≥ 30 kg/m2 as obese and BMI 25–30 kg/m2 as overweight) showed that there was no difference in the spontaneous abortion rate between overweight and obese groups. This is also the advantage of our analysis, because although researchers currently believe that high BMI will lead to a higher miscarriage rate in PCOS patients, it is still unclear whether higher BMI values will have worse impact on miscarriage. Our research shows that after reaching the limit of overweight (i.e., BMI ≥ 24 kg/m2 under the Chinese BMI standard or BMI ≥ 25 kg/m2 under the WHO standard), the spontaneous abortion rate of patients with PCOS has increased. However, after reaching the limit of obesity (i.e., BMI ≥ 28 kg/m2 under the Chinese BMI standard or BMI ≥ 30 kg/m2 under the WHO standard), the spontaneous abortion rate of PCOS patients is not statistically different from that of PCOS patients who only reach the standard of overweight (i.e., BMI 24–28 kg/m2 under Chinese BMI standards or BMI 25– 30 kg/m2 under WHO standards). It shows that the effect of high BMI on spontaneous abortion rate may be a process of qualitative change. This also provides clinicians with suggestions that for obese patients, the standard of BMI after weight loss should be as close as possible to the normal range in order to obtain ideal pregnancy results. Marquard et al. (51) found that PCOS women with high BMIs tended to have smaller oocytes than the control, but whether this can cause adverse pregnancy outcomes in PCOS patients remains to be determined. Khomami et al. (52). suggested that the combination of hyperandrogenism and IR and/or hyperinsulinemia may lead to adverse pregnancy outcomes in women with PCOS. High BMI may have a profound effect on the secretion and metabolism of sex hormones, resulting in changes in the bioavailability of estrogen and androgens and thereby affecting the normal development of follicles (53). As the degree of obesity increases, peripheral aromatization of androgens to estrogens improves, while liver synthesis of sex hormone-binding globulin (SHBG) decreases, which will result in increased levels of free estradiol and testosterone. This condition can be exacerbated by hyperinsulinemia, leading to further reduction and stimulation of ovarian androgen production by SHBG, excessive secretion of luteinizing hormone, and increased ratios of androgen to estrogen. These changes may disturb systemic endocrine environment, resulting in impaired follicle production and follicular atresia. Notably, the definitive mechanism of interactions between follicle generation and endometrial receptivity remains to be fully elucidated. Some authors (29) found that excessive administration of gonadotrophin during controlled ovarian stimulation due to a high body mass may also adversely affect embryo quality and endometrial receptivity, thereby leading to adverse pregnancy outcomes in overweight patients with PCOS.

Studies on gene expression in PCOS patients have found that the expression of multiple genes related to endometrial receptivity is decreased (54, 55). After investigating the endometrial transcriptome during the implantation window, Bellver et al. found that obese women have altered gene expression which is worsened in patients with PCOS (56). Furthermore, glucose metabolism plays an important role in the decidualization of endometrium during embryo implantation (57, 58). The uptake of glucose by cells in endometrium is mediated by glucose transporters (GLUT) - SLC2A family (59). It has been proven that SLC2A4 exists in human endometrium, and immunohistochemical studies suggest that it exists only in endometrial epithelial cells (60). As SLC2A4 is an insulin-dependent glutand obesity and PCOS are in a state of insulin resistance, Zhao et al. hypothesized that the decrease of SLC2A4 in endometrium would lead to endometrial insulin resistance and may damage endometrial metabolism (61). After further studying whether metformin could improve endometrial insulin resistance, they found that the expression of SLC2A4 mRNA and protein in obese PCOS patients was significantly lower than that in obese non-PCOS patients (61). After treatment with metformin for 3 months, the expression of SLC2A4 mRNA and protein was significantly improved (61). Other studies also found that the expression of SLC2A4 in obese PCOS patients was significantly lower than that in non-obese PCOS patients (60). As mentioned above, insulin resistance is also a metabolic feature of PCOS patients. It has been recognized that insulin can inhibit the production of IGFBP-1, a biomarker of decidualization (62). The study by Chang et al. also showed that embryonic development was not affected in insulin-resistant patients, but the endometrial receptivity was impaired (63). These mechanisms provide a basis for the treatment of metformin in PCOS patients. Several clinical studies have demonstrated that metformin can improve the status of hypergonadism and insulin resistance in PCOS patients with endometrial cancer and protect their fertility (64, 65). Compared with placebo, metformin significantly increased the ovulation rate (66). It is worth noting that the use of metformin before pregnancy may reduce the abortion rate (67, 68), but the evidence is insufficient. Other studies seemed to believe that the use of metformin has no significant impact on the abortion rate (69–72). Regarding the role of metformin in ovulation in infertile PCOS patients in the guidelines of the Practice Committee of the American Society for Reproductive Medicine, it has been pointed out that there is fair evidence that metformin used while attempting pregnancy and stopped at the initiation of pregnancy does not affect the rate of miscarriage (Grade B) and that there is insufficient evidence to recommend metformin during pregnancy to reduce the chance of miscarriage (Grade C) (73).

The effect of age was analyzed on the risk of spontaneous abortion in patients with PCOS undergoing ART. Although fewer articles were related to this issue with less representative results, it was interesting to find that patients with PCOS may have better pregnancy outcomes than those without PCOS at the same age. Patients without PCOS usually have fertility declined sharply when older than 35, but the IVF (*in vitro* fertilization) -related fertility declines moderately in patients with PCOS (28). This may be related to the number of oocytes and embryos available in patients with PCOS.

Subgroup analysis on chromosomal abnormalities revealed contradictory outcomes. Li et al. (31) considered that traditional G-banded chromosome karyotype analysis and/or fluorescence *in situ* hybridization had lower sensitivity than genome hybridization array test. The G-banded chromosome karyotype analysis is a morphological test based on naked eye recognition, with limited resolution and 5 to 10 Mb changes required (74), whereas the SNP array can detect a large area of changes from 1.89 to 16.00 Mb deletions or duplications and make up for the low resolution of the karyotype analysis (75). Chang et al. (76) compared the roles of single nucleotide polymorphism array (SNP array) and karyotype analysis in high-risk pregnant women prenatal diagnosis and found that the performance of the SNP array (11.3%) is significantly (p=0.039) better than that of the karyotype analysis (6.4%). However, more evidence is needed to prove whether minor chromosomal abnormalities could lead to different results.

In addition to analyses of the above factors, we tried to assess dyslipidemia because it might affect the spontaneous abortion rate of PCOS patients (29). However, the related articles were too limited for a meta-analysis. Dyslipidemia was defined as any one of the following criteria: serum cholesterol (TC)> 6.20 mmol/L, triacylglycerol (TG)> 2.25 mmol/L, low density lipoprotein (LDL)> 4.10 mmol/L, and high density lipoprotein (HDL) < I.03 mmol/L (29). Abnormal lipid metabolism may cause adverse pregnancy outcomes in patients with PCOS. Glueck et al. (77) found that the 4G polymorphism of the PAI-1 gene was more common in PCOS women than in the normal counterparts and was associated with high BMI and hyperinsulinemia complicated with hypertriglyceridemia. Sun et al. (78) proposed that homozygosity of ACE D or PAI-1 4G genes and complex carrier status were related to early pregnancy abortion. It was speculated that the 4G polymorphism of the PAI-1 gene may cause dyslipidemia to promote pathological changes and subsequently increase the abortion rate in patients with PCOS. However, Li et al. (29) found that patients with dyslipidemia, especially increased triacylglycerol levels, had higher BMI and increased gonadotropin during assisted reproduction dosage, resulting in an increase in the early abortion rate. Dyslipidemia can promote the development of IR, hyperandrogenism, oxidative stress, and anovulation in PCOS, increasing the risk of cardiovascular disease (79).

Dyslipidemia promotes pathological changes in patients with PCOS (80–87). It has been found that high visceral fat levels in patients with PCOS increased the fatty acid levels in circulating blood, which leads to increased lipolysis of circulating fatty acids in obese patients with PCOS, and this in turn impairs the role of insulin in adipose tissue and causes IR (80–83). In addition, dyslipidemia plays an important role in PCOS-associated inflammation (84), and high levels of free fatty acids may activate mononuclear cells to regulate the expression of chemokines and the release of cellular inflammatory factors in adipocytes. Apo-lipoprotein AI (Apo-AI) levels are reduced in the serum of patients with PCOS, and the Apo-AI content in granulosa cells proportionally changes the expression of steroidogenic enzymes, including CYP11A, 17-hydroxysteroid dehydrogenation Catalase (HSD), 3-HSD, and CYP19 (85). Decreased expression of CYP19 due to reduced Apo-AI may hinder subsequent conversion of testosterone to estradiol, leading to occurrence of hyperandrogenemia. Moreover, anovulation in PCOS patients was associated with abnormal blood lipid metabolism (86). High levels of triglycerides, free fatty acids, and oxidized LDLs (oxLDLs) in the serum can cause mitochondrial dysfunction while promoting release of reactive oxygen species, which ultimately contributes to ovarian damage and follicular atresia (87). Lectin-like oxLDL receptor-1 (LOX-1), toll-like receptor 4 (TLR4), and cluster of differentiation 36 (CD36) are oxLDL receptors. Activations of these oxLDL-dependent receptors can cause human granulosa cell (GC) apoptosis and ovulatory disorders. However, definitive relationship between dyslipidemia and adverse pregnancy outcomes in patients with PCOS remains to be elucidated because of insufficient research data.

It needs to be explained that there are two situations about pregnancy complicated with diabetes. In one condition, the patient’s glucose metabolism is normal before pregnancy, and diabetes only appears during pregnancy, which is called gestational diabetes mellitus (GDM). In the other condition, pregnancy is combined with pre-existing diabetes, which is also known as diabetes mellitus complicated with pregnancy. Hyperglycemia can cause abnormal embryonic development and even death, increasing the incidence of miscarriage (88). Patients with PCOS may have an increased risk of gestational diabetes (89). If the relationship between abnormal glucose metabolism status and miscarriage in patients with PCOS can be found, it will be of great help to clinicians for administration of preventive treatment.

Hyperglycemia first detected during pregnancy is classified as GDM. Although it can occur anytime during pregnancy, GDM generally affects pregnant women during the second and third trimesters. The oral glucose tolerance test (OGTT) for pregnant women is usually completed 24 weeks after pregnancy. However, many abortions in PCOS patients occur in the first trimester before 12 weeks, and few studies on abortion in PCOS patients would involve markers of glucose metabolism, such as fasting blood glucose (FBG) and oral glucose tolerance test 2h blood glucose (OGTT 2hBG). We did not find enough data to analyze in this study. Therefore, insulin, as a hormone that regulates human blood sugar levels, is often used to assess the level of glucose metabolism in patients, and a lot of studies had investigated insulin. That is why we chose homeostasis model assessment‐insulin resistance (HOMA-IR) as the metabolic markers of patients with PCOS.

In addition, our exclusion criteria involved drug administration (including metformin) or intervention for purposes other than assisted reproductive technology, and many patients with diabetes mellitus complicated with pregnancy have used medications. This is why we did not collect enough data to analyze this point of view, which is also one of the limitations of this article.

This is the first systematic review and meta-analysis of the risk factors that increase the spontaneous abortion rate in patients with PCOS diagnosed by using the Rotterdam criteria with ART. Other criteria like the National Institute of Health (NIH) criteria and Abdrigen Excess PCOS Society criteria are also used in the diagnosis of PCOS (2). The NIH definition uses the following two criteria to make the diagnosis: Chronic anovulation, Clinical and/or biochemical signs of hyperandrogenism (90). However, the NIH definition has the following limitations: difficulty to objectively measure the ovulatory dysfunction and to quantify both the clinical hyperandrogenism which may vary with different ethnic groups and the hypersensitivity of PCO morphology to ovarian stimulation. The Abdrigen Excess PCOS Society criteria is more focused on hypersexual hyperandrogenemia. Compared to patients with PCOS diagnosed according to the NIH criteria which defines PCOS as clinical and/or biochemical hyperandrogenism associated with ovulatory dysfunction (91), the Rotterdam criteria has a lower prevalence of impaired glucose tolerance or hyperinsulinemia (92). Therefore, one of the inclusion criteria for this meta-analysis was use of the Rotterdam criteria for the diagnosis of PCOS, which is generally accepted worldwide. Bias resulted from inconsistent diagnostic criteria was eliminated. Moreover, studies involving medications for overweight, hyperinsulinemia, and hyperandrogenemia were also eliminated, and the results obtained in this meta-analysis were thus objective. Because this systematic review and meta-analysis excluded patients with PCOS diagnosed according to criteria other than the Rotterdam criteria, the sample size was thus possibly reduced. In addition, fewer studies may have been retrieved regarding certain risk factors and may thus affect the effectiveness of the outcome. The current outcome of analysis can only represent the currently available studies, and further investigation is needed to confirm the outcome. Another limitation of this study was no hierarchical analysis on the influence of assisted reproductive technology. Studies (93, 94) have shown that the spontaneous abortion rate of assisted reproductive technology is significantly higher than that of normal pregnancy. However, the records about the impact of assisted reproductive technology on spontaneous abortion rate in patients with PCOS were too limited to be analyzed. All included studies in this report were related to assisted reproductive technology. In addition, since the included data were the original data mentioned in the literature rather than the adjusted OR value, there may be interference between risk factors, even though we had done the analysis of adjusted OR value about BMI, age, bLH, and bT in patients with PCOS. Small ample size is another limitation of this study.

Given the results of this systematic review and meta-analysis, it is suggested that patients with PCOS may reduce the spontaneous abortion rate by losing weight before preparing for pregnancy, and the ideal target of weight control is within the normal BMI range. Obese patients may not benefit much if they have lost some weight but remain in the overweight status. Furthermore, mitigating insulin resistance may also reduce the spontaneous abortion rate in PCOS patients undergoing ART. Preimplantation genetic testing may reveal some chromosomal abnormalities that cause miscarriage, but the optimal method remains to be elucidated.

## Data Availability Statement

The raw data supporting the conclusions of this article will be made available by the authors, without undue reservation.

## Author Contributions

G-MH contributed to the study design and critical revision of the manuscript. Y-FS, Z-YC, and Y-MX selected studies for inclusion and abstracted data. Y-FS did the statistical analyses and wrote the first draft. JZ, Y-MX, Y-ZW, and B-LG contributed to the study and revision of the manuscript. All authors contributed to the article and approved the submitted version.

## Funding

National Key Research and Development Project of China (2018YFC1002104, 2018YFC1003200, and 2017YFC1001004), Natural Science Foundation of Hebei Province in 2019(19JCZDJC6500(Z), and Taishan scholar project special funds (ts201712103).

## Conflict of Interest

The authors declare that the research was conducted in the absence of any commercial or financial relationships that could be construed as a potential conflict of interest.
