# Supplementary material for: High BMI and Insulin Resistance Are Risk Factors for Spontaneous Abortion in Patients With Polycystic Ovary Syndrome Undergoing Assisted Reproductive Treatment: A Systematic Review and Meta-Analysis
Source: Front Endocrinol (Lausanne). 2020 Dec 3;11:592495. doi: 10.3389/fendo.2020.592495 (PMC7744738; doi:10.3389/fendo.2020.592495)
Supplement: Supplementary file 1 [file DataSheet_1.docx]

Supplementary Materials

**Title: High BMI and insulin resistance are risk factors for miscarriage in patients with polycystic ovary syndrome: a systematic review and meta-analysis**

Yi-Fei Sun^1^, Jie Zhang^1^, Yue-Ming Xu^1^, Zi-Yu Cao^1^, Yi-Zhuo Wang^1^, Gui-Min Hao^1*^, Yu-Hua Shi^2*^, Bu-Lang Gao^1^

1.Department of Reproductive Medicine, the Second Hospital of Hebei Medical University, Shijiazhuang, Hebei, 050011 China

2.Center for Reproductive Medicine, Shandong University, China；Shandong Provincial Clinical Medicine Research Center for Reproductive Health, Jinan, 250100, China.

**Correspondence to:** Gui-Min Hao, Department of Reproductive Medicine, the Second Hospital of Hebei Medical University, Shijiazhuang, Hebei 050011, China. Email: [haoguimin@163.com](mailto:haoguimin@163.com)

**Supplementary Table 1:** **The PRISMA Checklist.**

| **Section / topic** | **#** | **Checklist item** | **Reported on page #** |
| --- | --- | --- | --- |
| **TITLE** | | |  |
| Title | 1 | Identify the report as a systematic review, meta-analysis, or both. | Title |
| **ABSTRACT** | | |  |
| Structured summary | 2 | Provide a structured summary including, as applicable: background; objectives; data sources; study eligibility criteria, participants, and interventions; study appraisal and synthesis methods; results; limitations; conclusions and implications of key findings; systematic review registration number. | Abstract |
| **INTRODUCTION** | | |  |
| Rationale | 3 | Describe the rationale for the review in the context of what is already known. | Introduction |
| Objectives | 4 | Provide an explicit statement of questions being addressed with reference to participants, interventions, comparisons, outcomes, and study design (PICOS). | Introduction |
| **METHODS** | | |  |
| Protocol and registration | 5 | Indicate if a review protocol exists, if and where it can be accessed (e.g., Web address), and, if available, provide registration information including registration number. | Methods |
| Eligibility criteria | 6 | Specify study characteristics (e.g., PICOS, length of follow-up) and report characteristics (e.g., years considered, language, publication status) used as criteria for eligibility, giving rationale. | Methods |
| Information sources | 7 | Describe all information sources (e.g., databases with dates of coverage, contact with study authors to identify additional studies) in the search and date last searched. | Methods |
| Search | 8 | Present full electronic search strategy for at least one database, including any limits used, such that it could be repeated. | Methods |
| Study selection | 9 | State the process for selecting studies (i.e., screening, eligibility, included in systematic review, and, if applicable, included in the meta-analysis). | Methods |
| Data collection process | 10 | Describe method of data extraction from reports (e.g., piloted forms, independently, in duplicate) and any processes for obtaining and confirming data from investigators. | Methods |
| Data items | 11 | List and define all variables for which data were sought (e.g., PICOS, funding sources) and any assumptions and simplifications made. | Methods |
| Risk of bias in individual studies | 12 | Describe methods used for assessing risk of bias of individual studies (including specification of whether this was done at the study or outcome level), and how this information is to be used in any data synthesis. | Methods |
| Summary measures | 13 | State the principal summary measures (e.g., risk ratio, difference in means). | Methods |
| Synthesis of results | 14 | Describe the methods of handling data and combining results of studies, if done, including measures of consistency (e.g., I^2^) for each meta-analysis. | Methods |

Page 1 of 2

| **Section/topic** | **#** | **Checklist item** | **Reported on page #** |
| --- | --- | --- | --- |
| Risk of bias across studies | 15 | Specify any assessment of risk of bias that may affect the cumulative evidence (e.g., publication bias, selective reporting within studies). | Methods |
| Additional analyses | 16 | Describe methods of additional analyses (e.g., sensitivity or subgroup analyses, meta-regression), if done, indicating which were pre-specified. | Methods |
| **RESULTS** | | |  |
| Study selection | 17 | Give numbers of studies screened, assessed for eligibility, and included in the review, with reasons for exclusions at each stage, ideally with a flow diagram. | Results |
| Study characteristics | 18 | For each study, present characteristics for which data were extracted (e.g., study size, PICOS, follow-up period) and provide the citations. | Results |
| Risk of bias within studies | 19 | Present data on risk of bias of each study and, if available, any outcome level assessment (see item 12). | Results |
| Results of individual studies | 20 | For all outcomes considered (benefits or harms), present, for each study: (a) simple summary data for each intervention group (b) effect estimates and confidence intervals, ideally with a forest plot. | Results |
| Synthesis of results | 21 | Present results of each meta-analysis done, including confidence intervals and measures of consistency. | Results |
| Risk of bias across studies | 22 | Present results of any assessment of risk of bias across studies (see Item 15). | Results |
| Additional analysis | 23 | Give results of additional analyses, if done (e.g., sensitivity or subgroup analyses, meta-regression [see Item 16]). | Results |
| **DISCUSSION** | | |  |
| Summary of evidence | 24 | Summarize the main findings including the strength of evidence for each main outcome; consider their relevance to key groups (e.g., healthcare providers, users, and policy makers). | Discussion |
| Limitations | 25 | Discuss limitations at study and outcome level (e.g., risk of bias), and at review-level (e.g., incomplete retrieval of identified research, reporting bias). | Discussion |
| Conclusions | 26 | Provide a general interpretation of the results in the context of other evidence, and implications for future research. | Discussion |
| **FUNDING** | | |  |
| Funding | 27 | Describe sources of funding for the systematic review and other support (e.g., supply of data); role of funders for the systematic review. | Funding |

*From:*  Moher D, Liberati A, Tetzlaff J, Altman DG, The PRISMA Group (2009). Preferred Reporting Items for Systematic Reviews and Meta-Analyses: The PRISMA Statement. PLoS Med 6(6): e1000097. doi:10.1371/journal.pmed1000097

For more information, visit: **www.prisma-statement.org**.

Page 2 of 2

**Supplementary Table 2: Search strategy and selected keywords**

Database: PubMed:

Search Strategy:窗体顶端

--------------------------------------------------------------------------------

 ((((((((((((((((polycystic ovary syndrome[MeSH Major Topic]) OR Ovary Syndrome, Polycystic[Title/Abstract]) OR Syndrome, Polycystic Ovary[Title/Abstract]) OR Stein-Leventhal Syndrome[Title/Abstract]) OR Stein Leventhal Syndrome[Title/Abstract]) OR Syndrome, Stein-Leventhal[Title/Abstract]) OR Sclerocystic Ovarian Degeneration[Title/Abstract]) OR Ovarian Degeneration, Sclerocystic[Title/Abstract]) OR Sclerocystic Ovary Syndrome[Title/Abstract]) OR Polycystic Ovarian Syndrome[Title/Abstract]) OR Ovarian Syndrome, Polycystic[Title/Abstract]) OR Polycystic Ovary Syndrome 1[Title/Abstract]) OR Sclerocystic Ovaries[Title/Abstract]) OR Ovary, Sclerocystic[Title/Abstract]) OR Sclerocystic Ovary[Title/Abstract])) AND ((((((((((((((((Abortion, Spontaneous[MeSH Major Topic]) OR Abortions, Spontaneous[Title/Abstract]) OR Spontaneous Abortions[Title/Abstract]) OR Spontaneous Abortion[Title/Abstract]) OR Early Pregnancy Loss[Title/Abstract]) OR Early Pregnancy Losses[Title/Abstract]) OR Loss, Early Pregnancy[Title/Abstract]) OR Losses, Early Pregnancy[Title/Abstract]) OR Pregnancy Loss, Early[Title/Abstract]) OR Pregnancy Losses, Early[Title/Abstract]) OR Miscarriage[Title/Abstract]) OR Miscarriages[Title/Abstract]) OR Abortion, Tubal[Title/Abstract]) OR Abortions, Tubal[Title/Abstract]) OR Tubal Abortion[Title/Abstract]) OR Tubal Abortions[Title/Abstract]) AND ( "1970/01/01"[PDat] : "2020/02/29"[PDat] )) (426)

Database: Embase <1970 January to 2020 February >

Search Strategy:窗体顶端

--------------------------------------------------------------------------------

1 ('polycystic ovary syndrome'/exp OR 'polycystic ovary syndrome') AND [1-1-1970]/sd NOT [21-3-2020]/sd (27,996)

2 'ovary polycystic disease' AND [1-1-1970]/sd NOT [1-3-2020]/sd (26,931)

3 'cystic ovary' AND [1-1-1970]/sd NOT [1-3-2020]/sd (156)

4 'micro polycystic ovary' AND [1-1-1970]/sd NOT [1-3-2020]/sd (18)

5 'ovary polycystic syndrome' AND [1-1-1970]/sd NOT [1-3-2020]/sd (3)

6 'ovary, polycystic' AND [1-1-1970]/sd NOT [1-3-2020]/sd 26,933

7 'polycystic ovarian disease' AND [1-1-1970]/sd NOT [1-3-2020]/sd (819)

8 'polycystic ovary' AND [1-1-1970]/sd NOT [1-3-2020]/sd (17,678)

9 'polycystic ovary disease' AND [1-1-1970]/sd NOT [1-3-2020]/sd (228)

10 'polycystic ovary syndrome' AND [1-1-1970]/sd NOT [1-3-2020]/sd (16,953)

11 'Stein Cohen Leventhal syndrome' AND [1-1-1970]/sd NOT [1-3-2020]/sd (1)

12 'Stein Leventhal disease' AND [1-1-1970]/sd NOT [1-3-2020]/sd (2)

13 'syndrome Stein Leventhal' AND [1-1-1970]/sd NOT [1-3-2020]/sd (11)

14 1 OR 2 OR 3 OR 4 OR 5 OR 6 OR 7 OR 8 OR 9 OR 10 OR 11 OR 12 OR 13 (28,293)

15 'spontaneous abortion' AND [1-1-1970]/sd NOT [1-3-2020]/sd (42,320)

16 'abortion, spontaneous' AND [1-1-1970]/sd NOT [1-3-2020]/sd (176)

17 'miscarriage' AND [1-1-1970]/sd NOT [1-3-2020]/sd (18,137)

18 'spontaneous abortion' AND [1-1-1970]/sd NOT [1-3-2020]/sd (42,320)

19 'early pregnancy loss' AND [1-1-1970]/sd NOT [1-3-2020]/sd (1,577)

20 'early pregnancy losses' AND [1-1-1970]/sd NOT [1-3-2020]/sd (240)

21 'loss, early pregnancy' AND [1-1-1970]/sd NOT [1-3-2020]/sd (4)

22 'losses, early pregnancy' AND [1-1-1970]/sd NOT [1-3-2020]/sd (1)

23 'pregnancy loss, early' AND [1-1-1970]/sd NOT [1-3-2020]/sd (13)

24 'pregnancy losses, early' AND [1-1-1970]/sd NOT [1-3-2020]/sd (3)

25 'miscarriages' AND [1-1-1970]/sd NOT [1-3-2020]/sd (7,833)

26 'abortion, tubal' AND [1-1-1970]/sd NOT [1-3-2020]/sd (5)

27 'abortions, tubal' AND [1-1-1970]/sd NOT [1-3-2020]/sd (4)

28 'tubal abortion' AND [1-1-1970]/sd NOT [1-3-2020]/sd (83)

29 'tubal abortions' AND [1-1-1970]/sd NOT [1-3-2020]/sd (15)

30 15 OR 16 OR 17 OR 18 OR 19 OR 20 OR 21 OR 22 OR 23 OR 24 OR 25 OR 26 OR 27 OR 28 OR 29 (49,076)

31 14 AND 30 (1,335)

Database: Web of Science

--------------------------------------------------------------------------------

TS= ((polycystic ovary syndrome OR Ovary Syndrome, Polycystic OR Syndrome, Polycystic Ovary OR Stein-Leventhal Syndrome OR Stein Leventhal Syndrome OR Syndrome, Stein-Leventhal OR Sclerocystic Ovarian Degeneration OR Ovarian Degeneration, Sclerocystic OR Sclerocystic Ovary Syndrome OR Polycystic Ovarian Syndrome OR Ovarian Syndrome, Polycystic OR Polycystic Ovary Syndrome 1 OR Sclerocystic Ovaries OR Ovary, Sclerocystic OR Sclerocystic Ovary) AND (Abortion, Spontaneous OR Abortions, Spontaneous OR Spontaneous Abortions OR Spontaneous Abortion OR Early Pregnancy Loss OR Early Pregnancy Losses OR Loss, Early Pregnancy OR Losses, Early Pregnancy OR Pregnancy Loss, Early OR Pregnancy Losses, Early OR Miscarriage OR Miscarriages OR Abortion, Tubal OR Abortions, Tubal OR Tubal Abortion OR Tubal Abortions)) Custom year range was 1970 to 2020 (1213)

**Supplementary Table 3:** **Quality assessment of cohort studies**

| NEWCASTLE - OTTAWA QUALITY ASSESSMENT SCALE COHORT STUDIES | | | | | | | | | |
| --- | --- | --- | --- | --- | --- | --- | --- | --- | --- |
| Study | Selection | | | | Comparability | Outcome | | | Quality score |
|  | Representativeness of the exposed cohort | Selection of the non exposed cohort | Ascertainment of exposure | Demonstration that outcome of interest was not present at start of study | Comparability of cohorts on the basis of the design or analysis | Assessment of outcome | Follow-up long enough for outcomes to occur | Adequacy of follow up of cohorts |  |
| Bailey,2014 | ☆ | ☆ | ☆ |  | ☆☆ | ☆ | ☆ |  | 7 |
| Weghofer ,2006 | ☆ |  | ☆ |  | ☆☆ | ☆ | ☆ |  | 6 |
| Cui,2016 | ☆ | ☆ | ☆ |  | ☆☆ | ☆ | ☆ |  | 7 |
| Hao,2017 | ☆ | ☆ | ☆ |  | ☆☆ | ☆ | ☆ |  | 8 |
| Huang,2014 | ☆ | ☆ | ☆ |  | ☆☆ | ☆ | ☆ |  | 7 |
| Landres,2010 | ☆ |  | ☆ |  | ☆☆ | ☆ | ☆ |  | 6 |
| Weber,2019 | ☆ | ☆ | ☆ |  | ☆☆ | ☆ | ☆ |  | 7 |
| Li，2019 | ☆ | ☆ | ☆ |  | ☆☆ | ☆ | ☆ |  | 8 |
| Li，2018 | ☆ |  | ☆ |  | ☆☆ | ☆ | ☆ |  | 6 |
| Li,2019 | ☆ |  | ☆ |  | ☆☆ | ☆ | ☆ |  | 6 |
| lin,2019 | ☆ | ☆ | ☆ |  | ☆☆ | ☆ | ☆ |  | 7 |
| Lu,2017 | ☆ |  | ☆ |  | ☆☆ | ☆ | ☆ |  | 6 |
| Ozgun,2011 | ☆ | ☆ | ☆ |  | ☆☆ | ☆ | ☆ |  | 7 |
| Pan,2018 | ☆ | ☆ | ☆ |  | ☆☆ | ☆ | ☆ |  | 7 |
| Qiu,2019 | ☆ | ☆ | ☆ |  | ☆☆ | ☆ | ☆ |  | 7 |
| Sheng,2017 | ☆ | ☆ | ☆ | ☆ | ☆☆ | ☆ | ☆ | ☆ | 9 |
| Wan,2018 | ☆ | ☆ | ☆ |  | ☆☆ | ☆ | ☆ |  | 7 |
| Wang,2016 | ☆ |  | ☆ | ☆ | ☆☆ | ☆ | ☆ |  | 7 |
| Yang,2018 | ☆ | ☆ | ☆ | ☆ | ☆☆ | ☆ | ☆ | ☆ | 9 |
| Cakiroglu,2017 | ☆ | ☆ | ☆ |  | ☆☆ | ☆ | ☆ |  | 7 |

**Supplementary Table 4: Quality assessment of control studies**

| NEWCASTLE - OTTAWA QUALITY ASSESSMENT SCALE CASE CONTROL STUDIES | | | | | | | | | |
| --- | --- | --- | --- | --- | --- | --- | --- | --- | --- |
| Study | Selection | | | | Comparability | Exposure | | | Quality score |
|  | Is the case definition adequate? | Representativeness of the cases | Selection of Controls | Definition of Controls | Comparability of cases and controls on the basis of the design or analysis | Ascertainment of exposure | Same method of ascertainment for cases and controls | Non-Response rate |  |
| Sawada,2015 | ☆ | ☆ |  | ☆ | ☆☆ | ☆ | ☆ |  | 7 |
| Lu，2016 | ☆ | ☆ |  | ☆ | ☆☆ | ☆ | ☆ |  | 7 |

**Supplementary Table 5. Characteristics of included studies**

| Author | Year | Nation | Sample size | Mean age | Mean BMI |
| --- | --- | --- | --- | --- | --- |
| Bailey et al. | 2014 | America | 51 | 32.24±3.3 | —— |
| Weghofer et al. | 2006 | Austrian | 35 | 36.5±4.1 | —— |
| Cui et al. | 2016 | China | 166 | 28.16±3.43 | —— |
| Hao et al. | 2017 | China | 75 | 29.07±3.42 | —— |
| Huang et al. | 2014 | China | 50 | 29.8±3.7 | 23.1±3.6 |
| Landres et al. | 2010 | America | 204 | 34.4±3.6 | 23.6±3.6 |
| Weber et al. | 2019 | America | 3232 | —— | —— |
| Li et al. | 2019 | China | 1636 | 30.73±3.15 | 22.46±3.03 |
| Li et al. | 2018 | China | 1271 | 31.5±3.3 | —— |
| Li et al. | 2019 | China | 328 | 29.1±2.8 | —— |
| lin et al. | 2019 | China | 944 | 32.96±3.55 | 24.47±1.90 |
| Lu et al. | 2017 | China | 144 | —— | 30.3±3.1 |
| Sawada et al. | 2015 | Japan | 64 | 31.8±4.4 | 24.3±5.1 |
| Ozgun et al. | 2011 | Turkey | 23 | 26.8±4.0 | —— |
| Pan et al. | 2018 | China | 305 | 29.36±3.17 | 23.58±1.47 |
| Qiu et al. | 2019 | China | 1468 | 30.11±3.56 | —— |
| Lathi et al. | 2014 | America | 78 | 35.71±4.24 | 23.3±4.75 |
| Sheng et al. | 2017 | China | 723 | 27.9±3.1 | —— |
| Wan et al. | 2018 | China | 184 | 29.3±3.4 | —— |
| Wang et al. | 2016 | China | 100 | 32.09±4.32 | 22 (18,31) |
| Yang et al. | 2018 | China | 58 | 26.69±3.09 | 22.76±1.55 |
| Cakiroglu et al. | 2017 | Turkey | 43 | 29.5±1.8 | —— |

**Supplementary Fig.1. Flow diagram of search strategy and selection process**


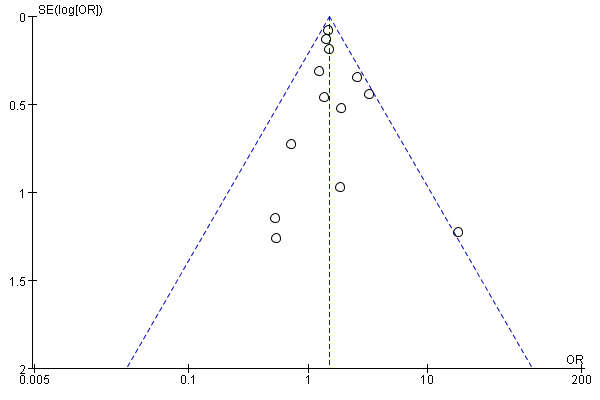
**Supplementary Fig.2. Assessment of risk of bias**


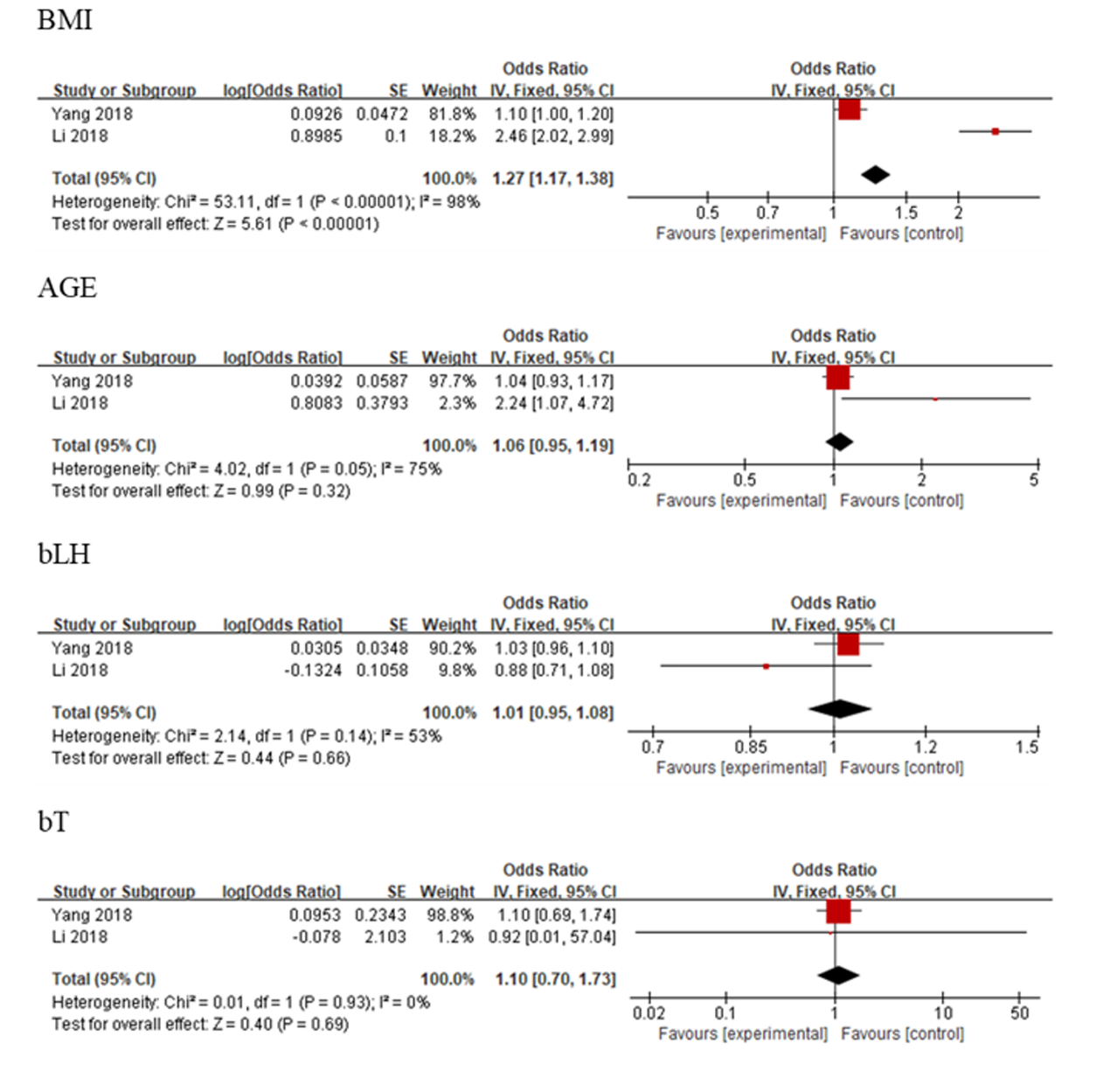


**Supplementary Fig. 3. Meta‐analysis for adjusted OR**
